# Ligamentous healing potential of the acromioclavicular ligament following acute anatomical reconstruction

**DOI:** 10.1007/s00402-021-03936-7

**Published:** 2021-05-20

**Authors:** L. R. Tuecking, B. Erdle, A. Bernstein, P. Ogon, M. Jaeger, N. P. Südkamp, K. Izadpanah, D. Maier

**Affiliations:** 1 Department of Orthopaedic Surgery, Hannover Medical School, Diakovere Annastift, Anna-von-Borries-Str. 1-7, 30625 Hannover-Kleefeld, Germany; 2 Department of Orthopaedics and Trauma Surgery, University Medical Center Freiburg, Hugstetter Strasse 55, 79106 Freiburg, Germany; 3 Center of Orthopaedic Sports Medicine Freiburg, Breisacher Str. 84, 79110 Freiburg, Germany

**Keywords:** Acromioclavicular joint, Acute dislocation, Dynamic horizontal instability, Acromioclavicular ligament, Ligament healing

## Abstract

**Background:**

Horizontal instability following acute acromioclavicular joint (ACJ) reconstruction still occurs with a high prevalence. Although the human acromioclavicular ligament complex (ACLC) represents the major horizontal ACJ stabilizer, experimental studies on healing characteristics are lacking. Therefore, the purpose of this histological study was to investigate the healing potential of the ACLC following acute anatomical reconstruction

**Methods:**

In this prospective clinical-experimental study, 28 ACLC biopsies were performed in patients with complete ACJ dislocations (Rockwood type 4 or 5) during acute hook plate stabilization (IG: implantation group; *n* = 14) and hook plate removal (EG: explantation group; *n* = 14). Histological analyses included Giemsa staining, polarized light microscopy and immunostaining against CD68, αSMA and collagen type I and type III. Histomorphological evaluation entailed cell counts, collagen expression score, ligament tissue maturity index (LTMI) and descriptive analysis of specific ligamentous structures. Statistics consisted of nonparametric Mann–Whitney *U* tests and a level of significance of *P* < .05.

**Results:**

Total cell counts (cells/mm^2^ 1491 ± 296 vs. 635 ± 430; *P* < 0.001) and collagen III expression (3.22 ± 0.22 vs. 1.78 ± 0.41; *P* < 0.001) were higher in EG compared to IG. Inversely αSMA + (11 ± 9 vs. 179 ± 186; *P* < 0.001) and CD68 + cell counts (56 ± 20 vs. 100 ± 57; *P* 0.009) were significantly lower in the EG. The EG revealed a comparable reorientation of ligamentous structures. Consistently, ACLC samples of the EG (21.6 ± 2.4) displayed a high total but differently composed LTMI score (IG: 24.5 ± 1.2; *P* < 0.001).

**Conclusions:**

This experimental study proved the ligamentous healing potential of the human ACLC following acute anatomical reconstruction. Histomorphologically, the ACLC reliably showed a ligamentous state of healing at a mean of about 12 weeks after surgery. However, processes of ligamentous remodeling were still evident. These experimental findings support recent clinical data showing superior horizontal ACJ stability with additional AC stabilization in the context of acute ACJ reconstruction. Though, prospective clinical and biomechanical studies are warranted to evaluate influencing factors on ACLC healing and potential impacts of acute ACLC repair on clinical outcome.

**Study type:**

Controlled Laboratory Study

## What this study adds to existing knowledge

The clinical explanation for ACL healing is an assumption of ligamentous stump scarring. Despite the relatively high prevalence of clinical impairment due to persistent horizontal instability, no study so far has examined the tissue properties to our knowledge. This would be the first step when looking for ways to improve outcome. To our knowledge, this study is the first to demonstrate histomorphological healing of the human ACLC following acute acromioclavicular joint reconstruction. In this present study, the ACLC showed ligament healing properties with comparable reorientation and composition of ligamentous structures in surgically treated ACLC (with AC cerclage) when compared to intact ACLC structures within acute ruptured specimen. These results suggest that operative treatment of acute ACLC ruptures (AC cerclages) lead to intrinsic ligament-like healing of the ACLC.

## Introduction/Background

The acromioclavicular ligament comprises an anatomical complex of 4 ligaments for circumferential enforcement of the acromioclavicular capsule anteriorly, posteriorly, inferiorly and superiorly. Biomechanically, the acromioclavicular ligament complex (ACLC) functions as the major stabilizer of the acromioclavicular joint (ACJ) [1–4]. The superior acromioclavicular ligament (SACL) with its dominant superoposterior bundle represents the strongest portion of the ACLC.

Direct and indirect impacts on the lateral shoulder are regarded as typical injury patterns for acute ACJ dislocation, which represents one of the most common shoulder injuries in younger and active populations [5, 6]. Ligamentous ACJ stabilizers rupture with progressive force following a specific sequence (acromioclavicular ligament, coracoclavicular ligaments and deltotrapezoidal fascia). Nevertheless, the radiographic classification of ACJ instability as described by Rockwood is still most common [7]. Type I and II injuries are usually successfully treated conservatively [1, 8–10]. While optimal treatment of type III injuries is still controversial, complete ACJ dislocations (type IV injuries and higher) may qualify for surgical reconstruction [8, 11]. However, persistent dynamic horizontal instability (DHI) has been reported in up to 50% of cases following both arthroscopic and open acute ACJ reconstruction [12–15]. Additionally, DHI has been associated with inferior functional outcomes in several studies [12, 16, 17]. Therefore, clinically relevant DHI should be excluded even in lower grades of ACJ instability [18].

Insufficient intrinsic healing of the ACLC is assumed to be the main causation of persistent symptomatic DHI. Multiple histomorphological studies have investigated the healing response of the anterior cruciate ligament (ACL) and medial collateral ligament (MCL) of the human knee joint following acute rupture [19–23]. These studies confirmed that ligament healing progresses in typical overlapping phases of inflammation, proliferation and remodeling but shows fundamental differences between intra- and extraarticular ligaments. Recently, a fundamental histological study described the dynamic healing responses of the ACLC following acute traumatic rupture [24]. However, the authors are not aware of any study investigating the intrinsic healing characteristics of the human ACLC. Therefore, this fundamental, histological study aims to investigate the intrinsic healing potential of the human ACLC following acute anatomical reconstruction.

## Materials and methods

This study was approved by the Ethics Committee of the University of Freiburg (Vote-Nr.: 490/13), and written informed consent was obtained from all participating patients. From 06/2014 to 04/2016, the study consecutively included a total of 28 patients with complete, acute ACJ dislocations (type 4 or 5 according to the Rockwood classification; *n* = 14) and patients with an equal injury who had undergone surgical reconstruction via hook plate implantation (*n* = 14). The inclusion and exclusion criteria are listed in Table 1.

**Table 1:** Inclusion and exclusion criteria

Inclusion criteria	Exclusion criteria
Aged from 18 to 60 years	Incomplete ACJ dislocations (< Rockwood type IV)*
Isolated, acute ACJ dislocation (≥ Rockwood type IV)*	Nonanatomic ACJ reduction following acute ACJ reconstruction (hook plate implantation)*
Surgery required due to:	Incomplete documentation of time intervals
Acute ACJ reconstruction (hook plate implantation) Hook plate removal (anatomic ACJ reduction proven with follow-up radiographs)	Radiological signs of ACJ degeneration/osteoarthritis
Full legal competence	History of previous ipsilateral ACJ injury or surgery
	Comorbidities with potential impairment of ligament healing (e.g. malignancies, immunosuppression, diabetes, acute or systemic infections)

^*^Radiographic evaluation and grading following guidelines by Martetschläger et al.[25]

### Patient inclusion

During the study period, 28 of 37 eligible patients were included, corresponding to an inclusion rate of 80%. Noninclusion was caused by lack of clear orientation due to insufficient marking or segmentation of specimens (*n* = 7) and incomplete documentation (lack of time interval information, *n* = 2). ACJ dislocations treated operatively within 3 weeks (≤ 21 days) after trauma were defined as acute dislocation.

### Surgical technique

**Fig. 1:**
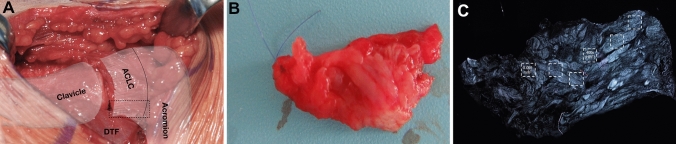
Illustration of biopsy location, macro- and microscopic sample presentation. Intraoperative situs of the acromioclavicular joint and the location of biopsy (dotted rectangle, **a**). Macroscopic view of a sample with suture mark of the rupture zone (**b**). Microscopic picture of a sample of the explantation group (EG) with polarized light microscopy (**c**) and exemplary definition of ROIs. Smaller boxes are representing the regions of interest (ROIs), defined as a high-power field (HPF) with 400 × magnification and an area of 0.0095 mm^2^

Surgeries were performed or supervised by 2 experienced shoulder surgeons (DM, KI). ACLC biopsies were harvested during acute ACJ stabilization (Implantation Group, IG) or during hook plate removal approximately 3 months later (Explantation Group, EG). The operative technique complied with a standardized protocol described previously [26]. Full-thickness, longitudinal tissue samples were taken from the superoposterior bundle of the ACLC (length: 1.5–2.5 cm × width: 0.4–0.7 cm; Fig. 1a). To guarantee the reproducibility of orientation within each histological specimen, the samples were detached subperiosteally at their intact bony insertion, and the rupture zone was marked with sutures (Fig. 1b). Anatomical ACLC repair was performed using transosseous and/or directly absorbable sutures. No coracoclavicular ligament reconstruction was performed. All patients strictly followed a standardized postoperative rehabilitation protocol that excluded abduction/elevation > 90°, heavy lifting, push-up movements and forced adduction until removal of the hook plate. Standardized radiographs (AP, transaxillary view) were obtained 6 weeks postoperatively to verify anatomic ACJ reduction.

### Tissue preparation

The tissue samples were immediately processed further for cryopreservation. They were flash-frozen (Tissue-Tek, O.C.T.) and cut longitudinally (7 μm width) using a cryostat. Giemsa staining and immunostaining (ZytoCHEM-Plus HRP Polymer-Kit, ZYTOMED Systems) were performed after formalin fixation with antibodies against alpha-smooth muscle actin (αSMA) as a myofibroblast marker (Abcam; ab7817; 1:100), cluster of differentiation 68 (CD68) as a marker for macrophages (DakoCytomation; M0718; 1:50), collagen type I (Abcam; ab6308; 1:100) and collagen type III (Abcam; ab7778; 1:500).

### Regions of interest

For obvious ethical reasons, it was not feasible to employ a control group consisting of native, healthy human ACLC biopsies. Thus, specimens of the intact zone of acutely ruptures ACLCs represented the “best available” control group. These specimens were obtained during hook plate implantation for acute ACJ reconstruction and were labeled as “Implantation Group” (IG). The ACLC biopsies obtained during hookplate explantation were regarded to represent the healed state of the ACLC, and were defined as “Explantation Group” (EG). The “Regions of Interest” (ROI) for every tissue sample were defined by three investigators (LT, DM, KI) using a consensus process based on standardized criteria. Within intact zones of acutely ruptured ACLC tissue specimens (IG), 3 ROIs were evenly placed upon morphologically regular ligamentous crimp structures. Within samples of the EG, 6 ROIs were evenly placed upon collagenous structures over the longitudinal course of the ligament to ensure proportionate evaluation of the full extension of the ligament (Fig. 1c). One ROI comprised an image of the tissue at 400 × magnification and therefore corresponded to one high-power field (HPF, ROI ≜ HPF) or an area of 0.095 mm^2^.

### Histological evaluation

For the histological examination average values (cell number/mm^2^) of all ROIs were determined for every tissue sample.

Histological evaluation (LT, DM, KI) of the tissue samples was performed by means of quantitative, semiquantitative and morphological analyses.

For quantitative analysis, the number of all cells, fibroblasts and fibrocytes in each HPF were counted using the 'Cell Counter' plugin in the Fiji software (ImageJ, v. 2.0.0, Open Source). A high occurrence of tissue cells, expressed by number of all cells/total cell count is generally valued as a non-specific sign of the early (“granulation”) phase in the healing process and a progress marker of remodelling processes. The relation of fibrocytes to fibroblasts is a marker of tissue maturity within ligament healing processes (as analyzed in the LTMI, see below). Alpha smooth muscle actin as well as CD68 are immunohistochemical marker for activated myofibroblasts and respectively, monocyte lineage cells (circulation and resident macrophages). These cell types are known to play a significant role in early ligament healing phases, and are known to decrease in number in later phases of ligament healing.

The semiquantitative analysis of a tissue sample consisted of collagen expression scoring and determination of the Ligament Tissue Maturity Index (LTMI).

The intensity of collagen staining in each HPF was assessed with a modified Remmele score [19] ranging from 0 (no expression/no immunohistochemistry (IHC)-staining density) to 4 (high expression/high IHC-staining density). The LTMI [27], with a maximum score of 28 points for the assessment of ligament maturity, is based on the following subscores: (1) the cellular subscore (0–10): cell density, nuclear aspect, orientation and formation; (2) the collagen subscore (0–12): bundle width and orientation, crimp length; and (3) the vascularity subscore (0–6): vessel density, orientation and maturity. A high LTMI score is valued as a high ligament tissue maturity after healing processes (native ligament-like histological appearance).

For morphological analysis, cellular shape and orientation, collagenous structure, collagenous tissue orientation and vascularity were described based on the evaluation process of Murray et al. [27].

### Statistical evaluation

Statistical evaluation was performed using Microsoft Excel 2013 and GraphPad Prism 6 software (GraphPad Software, USA). A detailed, descriptive histomorphological intergroup comparison was performed. Semiquantitative and quantitative comparisons were based on mean values and standard deviations (± SD). Due to the relatively small sample size without a normal distribution of data, nonparametric statistical tests were applied. Statistical significance was detected with the Mann–Whitney *U* Test. The level of statistical significance was set at *P* ≤ 0.05.

## Results

The group-specific demographic parameters are displayed in Table 2. Patient age did not significantly differ between the groups (*P* = 0.992). Additionally, there were no significant variations related to the distribution of sex or type of ACJ dislocation between the groups (*P* = 1.000; *P* = 0.622). The time intervals between trauma and initial surgery showed no significant difference between ACLC samples in the EG and IG (*P* = 0.623).

**Table 2:** Group-specific demographic parameters

	Implantation group	Explantation group	*P* value
Number of acquired tissue samples	14	14	1.000
Mean patient age (years)	37.0 (± 10.9)	37.8 (± 12.1)	0.992
Patient sex			1.000
Male	14	14	
Female	0	0	
ACJ dislocation			0.622
Rockwood grade IV	2	3	
Rockwood grade V	12	11	
Time interval trauma—initial surgery (days)	11.33 (± 6.5)	10.0 (± 6.5)	0.623
Time interval hook plate implantation—biopsy (days)	–	85.54 (± 17.3)	

Mean (± standard deviation), *ACJ* acromioclavicular joint

### Quantitative analysis

**Fig. 2:**
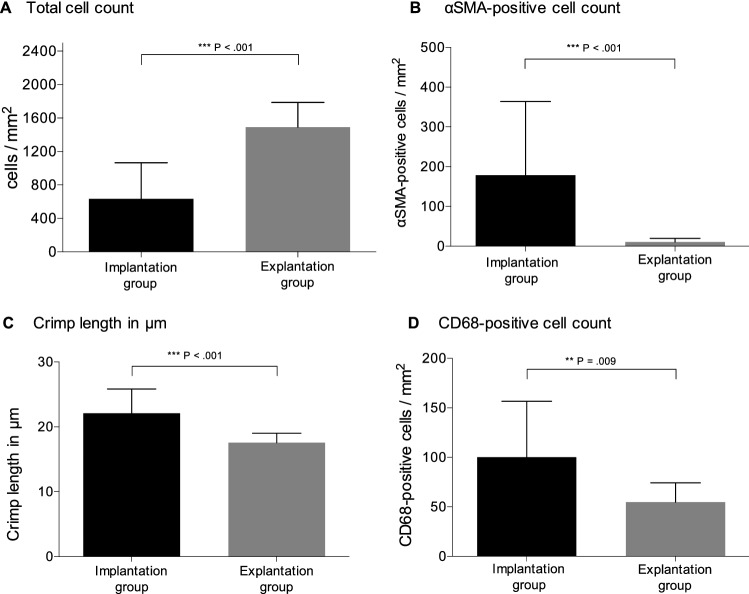
Histological quantitative results of cellular analysis. Quantitative histological measurements are shown as the mean (bar) with standard deviation (error bar). Total cell count (**a**), alpha-smooth muscle actin (αSMA)-positive cell count (**b**), crimp length (**c**), and CD68-positive cell count (**d**)

Quantitative results are summarized and displayed in Table 3 and Fig. 2.

**Table 3:** Quantitative and semiquantitative analysis of groups

Groups	Implantation group	Explantation group	*P* value
Total cells/mm^2^	635 ± 431	1491 ± 296	< 0.001
αSMA^+^- cells/mm^2^	179 ± 186	11 ± 9	< 0.001
CD68^+^- cells/mm^2^	100 ± 57	55 ± 20	0.009
Crimp length in µm	22.1 ± 3.8	17.6 ± 1.5	< 0.001
Collagen type I expression score	2.1 ± 0.72	2.2 ± 0.67	0.871
Collagen type III expression score	1.8 ± 0.41	3.2 ± 0.35	< 0.001

Mean value ± standard deviation (SD), *αSMA* alpha smooth muscle actin

Total cell counts in the EG were 2.3-fold higher than those in the IG (P < 0.001). Immunohistochemical analysis showed significant lower immunoreactivity in the EG with lower αSMA^+^ (16-fold) and CD68^+^ (1.8-fold) cell counts (*P* < 0.001, *P* = 0.009). ACLC samples in the EG exhibited a smaller crimp length (18 ± 1 µm), with a 1.2-fold decrease in crimp length compared to the IG samples (22 ± 4 µm; *P* = < 0.001).

### Semiquantitative analysis

**Fig. 3:**
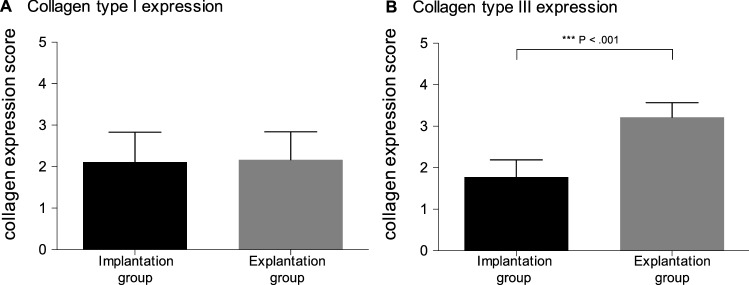
Histological semiquantitative results of collagen scoring. Semiquantitative histological measurements are shown as the mean collagen expression score (bar) with standard deviation (error bar). Collagen type I expression (**a**) and collagen type III expression (**b**)

Figure 3 illustrates group-specific expression scores for collagen types I and III, whereas semiquantitative results are summarized in Table 3. Although the expression scores of collagen type I did not differ between both groups (*P* = 0.871), the expression of collagen type III was significant lower within EG samples in comparison to IG samples (*P* < 0.001).

**Fig. 4:**
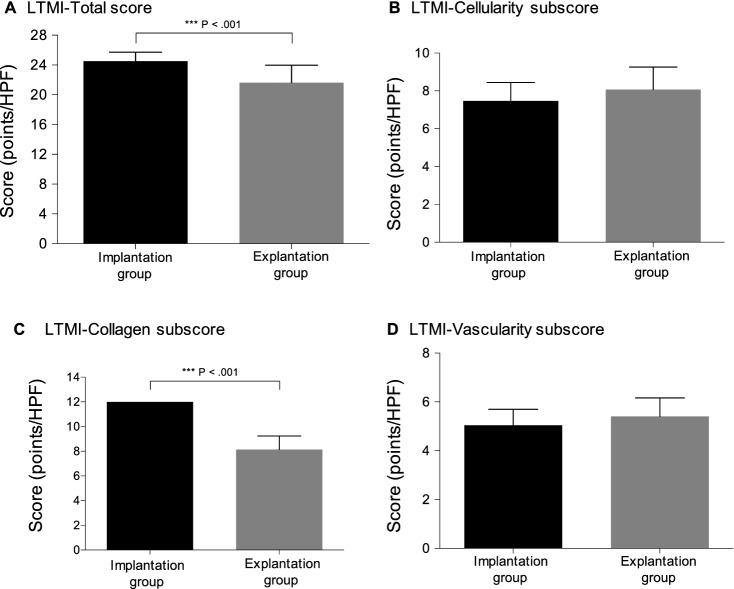
Histological semiquantitative measurement of the Ligament Tissue Maturity Index (LTMI). Total LTMI score (**a**), cellularity subscore (**b**), collagen subscore (**c**), vascularity subscore (**d**). Mean score (bar) with standard deviation (error bar)

Table 4 and Fig. 4 summarize and display the group-specific total score and subscores of the LTMI.

**Table 4:** Semiquantitative analysis of ligament tissue maturity index (LTMI)

**Groups**	Implantation group	Explantation group	*P* value
Total LTMI score	24.5 (± 1.2)	21.6 (± 2.4)	< 0.001
Cellularity subscore	7.5 (± 1.0)	8.1 (± 1.2)	0.134
Collagen subscore	12.0 (± 0.0)	8.1 (± 1.1)	< 0.001
Vascularity subscore	5.1 (± 0.7)	5.4 (± 0.8)	0.127

Ligament tissue maturity index (LTMI) scores and subscores are shown as the mean (± standard deviation)

IG samples and EG samples both showed high total LTMI scores (*P* = < 0.001) but with a significantly different composition. Group-specific cellularity and vascularity subscores did not differ (*P* = 0.134, *P* = 0.127 respectively). The collagen subscore of the EG, however, revealed a significant decrease (8.1 ± 1.1 points; *P* < 0.001) compared to the IG specimens, which scored a maximum of 12.0 (± 0) points.

### Morphological analysis

#### Cellularity

**Fig. 5:**
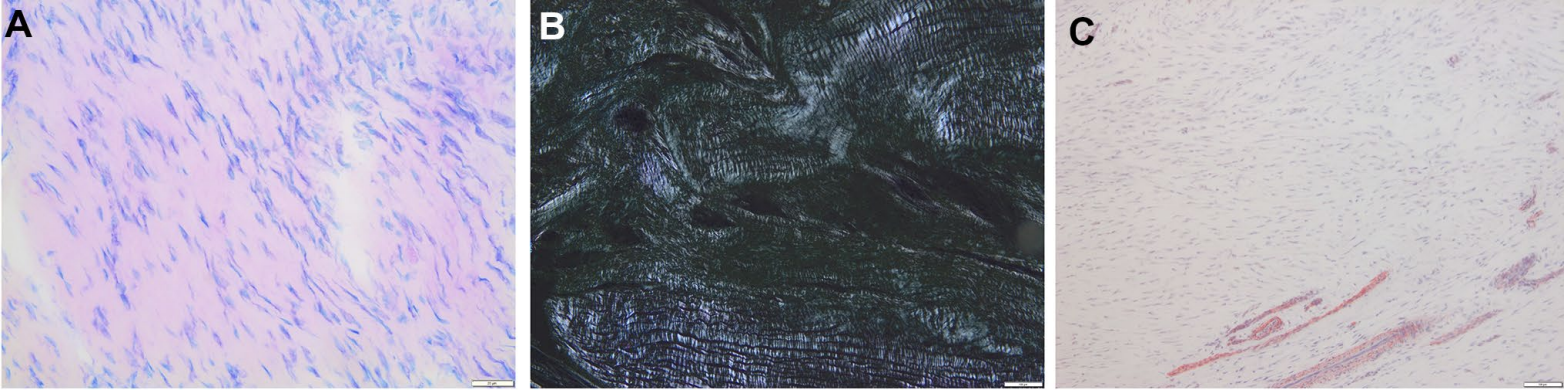
Histomorphological characteristics of healed ACLC samples. Explantation group (EG) samples show clear alignments of cell nuclei (**a**), collagen fibers (**b**) and vessels (**c**) along the long axis of the ligament. Polarized light microscopy shows homogenous crimp formation (**b**). Microscopic images of Giemsa staining (**a**) at 400 × magnification (scale bar 50 µm), polarized light (**b**) and αSMA-positive staining (**c**) at 100 × magnification

The most common cell shape found in IG samples was spheroid to fusiform, and the cell type was consistent with activated fibrocytes. Cells were aligned in rows or columns following the collagen structure (longitudinal axis) of the ligament. In EG samples, fibrocytes with fusiform cell nuclei were predominant (Fig. 5). Cells in EG samples were oriented along the ligament axis but lacked organization in rows and columns.

#### Collagen structure

IG samples showed a homogenous crimp structure and mainly orientated collagen bundles with thin endo- and epiligamentous tissue layers. Distinct endo- or epiligamentous tissues could not be identified in EG samples. Crimp structures were nonhomogeneous in the EG. Collagen bundles in EG samples displayed a clear axial orientation.

#### Vascularity

A lower vessel density was found in IG samples showing thin arterioles with a clear axial alignment within the ligament. EG samples showed a higher density and rather diffuse distribution of vessels with a predominantly longitudinal orientation.

## Discussion

The main finding of this experimental study was that the human acromioclavicular ligament disposes of ligamentous healing potential following acute anatomical reconstruction.

Operative treatment for acute ACJ dislocation should follow the principle of anatomic reduction and stabilization of all injured ligaments to enable their best possible intrinsic healing [12, 25, 28]. In view of the high incidence of acute ACJ injuries, the lack of basic knowledge of ligamentous healing characteristics is rather surprising [6, 29]. In contrast, ligament healing following rupture and operative treatment in ligaments of the knee (MCL, ACL) have been studied quite extensively [19–21, 23, 30]. Based on these fundamental studies, distinct healing phases of ligaments could be defined. Early healing responses for example consist of inflammation and proliferation with the formation of scar tissue, providing the structural basis for ligament regeneration. This phase of remodeling with the rearrangement of matrix elements, however, shows fundamental variations between intraarticular (e.g., ACL) and extraarticular (e.g., MCL) ligaments [20, 21, 31, 32]. Histologically, ligament remodeling is characterized by the conversion of collagen type III to collagen type I, a decrease in the total cell count, a decrease in vascular density and axial realignment of collagen fibers [20, 21, 32]. In a rabbit model for example, MCL remodeling was evident as early as 14–30 days post injury by decreased cellularity and increased alignment of collagen structure [20, 32]. In contrast, the total cell count in intraarticular ligaments (e.g. ACL) was still 3.2-fold higher 16 to 20 weeks postinjury, and a decrease in cellularity indicating completion of ligament remodeling did not occur until 52 to 104 weeks. In the present study, ACLC samples of the EG still exhibited evidence of scar tissue formation with high cell densities and strong collagen type III expression levels at three months post-operative. Compared to samples in the IG, EG samples harvested at a mean of 12 weeks after surgery showed a 2.3-fold higher cellularity and a 1.78-fold higher collagen type III expression. Therefore, it might be concluded that EG samples defined as partly intraarticular ligament complex were still subjected to remodeling at the investigated time point of implant removal. Additionally, EG samples showed a significantly shorter crimp length, which was also observed and evaluated as a remodeling process in other histological ligament healing studies [33]. In contrast to IG samples, EG samples showed a low immunoreactivity to CD68 and αSMA. Cells with αSMA positivity play a crucial role in the early healing response and early remodeling phase of ligaments [19, 23, 31]. As found in histological studies of MCL healing, decreases in αSMA-positive cell counts occur after the early remodeling phase (3–6 weeks) [31, 34]. Accordingly, EG samples displayed a distinctly low density of activated myofibroblasts. This finding might lead to the assumption that EG samples had already passed the early phase of remodeling and ligament healing at a mean of 3 months after acute reconstruction.

CD68 protein is expressed by monocyte lineages as well as circulating and resident macrophages. Macrophages play a critical role during the early inflammatory response with the expression of cytokines and chemotaxis of specific cell types to initiate processes of ligament healing. However, prolonged activity of CD68-positive cells can impair ligament healing and remodeling. Therefore, CD68-positive cells should not be overrepresented during later healing phases [34, 35]. Consistently, the low CD68 immunoreactivity in EG samples could be regarded as a sign for ligament remodeling and healing.

On a structural basis, the histomorphological analysis mainly showed an alignment of collagenous structures, cells and vessels following the long-axis of the ligament resulting in a high maturity score of the EG samples. Subscores of cellularity and vascularity already showed comparable scores between EG and IG samples. Nevertheless, a significant decrease of crimp length and sporadic presence of malaligned collagen bundles lead to a decreased collagen subscores resulting in a significant lower total LTMI score in EG samples. However, the total LTMI score of EG samples had already reached about 90% of the IG samples.

In conclusion, present findings suggest that acute anatomical reconstruction of ACLC ruptures reliably leads to intrinsic ligamentous healing. A recent experimental study of the ACLC healing response defined the first 3 weeks after trauma as the acute phase [24]. Histomorphologically, reparative changes peaked as early as within the second week after ACLC rupture. Therefore, the authors concluded that surgical treatment of acute ACJ dislocations ideally should be performed as early as possible to exploit the utmost ligamentous healing potential. In the present study, acute operative repair was performed within the second week at an average time point of 10 days after trauma. In accordance with these experimental findings, clinical data support the existence of intrinsic ACLC healing. The use of an additional AC cerclage during ACJ reconstruction procedures led to improved horizontal ACJ stability [36]. Another prospective MRI-based case series showed ACLC healing in all cases 16 months after double-bundle CC reconstruction combined with an additional AC cerclage [37]. However, experimental data on human ACLC healing did not exist so far.

Certainly, this experimental study entails specific limitations. The total study population (*n* = 28) was limited, thereby decreasing the statistical power. It might also be prone to a gender bias, as all subjects were male. Time intervals from injury to surgical repair are comparable within both groups, yet may still affect the healing response. Appropriate subgroup analyses were not possible due to small study population. For obvious ethical reasons, ACLC tissue samples could not be taken from healthy individuals. Therefore, the best available control group for this study consisted of specimens of acutely ruptured ACLCs. These IG samples were obtained from the intact insertion zone opposite of the rupture zone. Total LTMI scores in the IG showed high scoring values indicating a native-like status of these samples. However, timing of obtaining the IG samples within an acute state might influence immunoreactivity of αSMA- and CD68 since these cell types are evident in hyperacute healing phases. Finally, this experimental study did not intend to correlate the histological results with clinical outcomes. However, no patient showed clinical or radiological signs of persistent ACJ instability before, during or after implant removal.

## Conclusion

This experimental study proved the ligamentous healing potential of the human ACLC following acute anatomical repair. Histomorphologically, the ACLC reliably showed a ligamentous state of healing at a mean of about 12 weeks after surgery. However, processes of ligamentous remodeling were still evident at this time point. These experimental findings support recent clinical data showing superior horizontal ACJ stability after additional AC stabilization in the context of acute ACJ reconstruction. Though, prospective clinical and biomechanical studies are warranted to evaluate influencing factors on ACLC healing and impacts of acute ACLC repair on clinical outcome.
